# Understanding Organic Adlayer Formation on CoOx-Terminated PtCoO_2_

**DOI:** 10.3390/nano16201296

**Published:** 2026-10-13

**Authors:** Kerry Hazeldine, Jean-Philippe Soulié, Marko Vehkamäki, Mikko Ritala, Christoph Adelmann, Silvia Armini

**Affiliations:** 1 IMEC, 3001 Leuven, Belgium; jean-philippe.soulie@imec.be (J.-P.S.); christoph.adelmann@imec.be (C.A.); silvia.armini@imec.be (S.A.); 2 Department of Chemistry, University of Helsinki, 00560 Helsinki, Finland; marko.vehkamaki@helsinki.fi (M.V.); mikko.ritala@helsinki.fi (M.R.)

**Keywords:** PtCoO_2_, delafossite oxides, self-assembling monolayers, organic layer, passivation

## Abstract

PtCoO_2_ is an attractive candidate for next-generation interconnects due to its exceptionally low in-plane resistivity, but its susceptibility to degradation under reducing conditions necessitates effective surface passivation strategies. In this work, the deposition of organic adlayers on PtCoO_2_ was systematically investigated using octadecylphosphonic acid (ODPA), octylphosphonic acid (OPA), 1-octadecanethiol (ODT), and trifluoroacetic acid (TFA). The influence of immersion time (0.5–48 h) and surface pre-treatment (untreated, ethanol-cleaned, and UV-ozone-treated surfaces) was evaluated using water contact angle (WCA), X-ray photoelectron spectroscopy (XPS), and atomic force microscopy (AFM). Low-energy ion scattering (LEIS) confirmed a CoOx-rich surface termination, which strongly influenced adsorption behaviour. Among the molecules studied, phosphonic acids exhibited the strongest interaction with the surface, with ODPA producing the highest WCAs (97–99°) and clear evidence of phosphonate bonding. Comparison with OPA revealed the importance of alkyl chain length in promoting hydrophobicity and molecular organisation, yielding values of 77–88°. In contrast, ODT exhibited heterogeneous adsorption, sulphur oxidation, and aggregate formation, while TFA showed limited surface retention. Overall, ODPA was identified as the most effective adsorbate, establishing phosphonic acid-based self-assembled monolayers as promising candidates for the passivation of PtCoO_2_ and related conductive oxide materials.

## 1. Introduction

As devices continue to scale towards the sub-10 nm metal-pitch dimensions, copper is no longer meeting the line-resistivity requirements due to high grain-boundary and surface scattering, leading to high resistive-capacitive delay and energy dissipation [1,2,3,4]. In addition, copper also requires adhesion and barrier liners, which, at decreasing scales, occupy an increasing relative volume [5].

The conductive delafossite oxide, PtCoO_2_, is a promising alternative owing to its ultra-low anisotropic in-plane resistivity, with a value of 2.1 μΩ cm [6]. For device scaling applications, PtCoO_2_ offers the advantage of a much lower bulk resistivity compared to other leading alternatives such as Ru or Mo [7,8,9]. The anisotropic in-plane resistivity also offers an advantage for interconnect applications if the correct crystallographic orientation is achieved; surface, interface, and grain boundary scattering can be suppressed [10,11].

The general formula of delafossite oxides is ABO_2_, where A and B are both transition metals, with A frequently being noble metals. Delafossite oxides exist in an alternative layered rocksalt structure where the ‘A’ atoms are arranged in a triangular lattice, whilst the ‘B’ atoms are configured in a BO_6_ octahedral layer within a triangular lattice [12,13].

Despite PtCoO_2_‘s exceptional electrical properties, its practical implementation is currently critically limited by its susceptibility to material degradation under reducing environments. In particular, in our recent work, we demonstrated that forming-gas annealing of PtCoO_2_, a standard and common process during device integration, leads to oxygen loss and metal reduction, resulting in a significant increase in sheet resistance [14]. The significant degradation in functional performance observed highlights the urgent need for effective strategies to stabilise and protect the material under processing conditions.

Surface passivation via the deposition of organic adlayers was investigated due to the many advantages offered by organic coatings, including chemical tunability, conformal coverage, the ability to tailor interfacial properties without fundamentally altering the bulk characteristics of the underlying material, reduced interface effects, and low influence on the electrical resistivity of the PtCoO_2_. The effectiveness of organic passivation is reliant on control of the deposition parameters, including the choice of organic species, the deposition times, and the surface pre-treatment conditions, to name a few [15,16]. To date, a comprehensive and systematic optimisation of these parameters for PtCoO_2_ has not been reported.

Previous studies have demonstrated phosphonic acid self-assembled monolayers on conventional oxide substrates such as ZnO and TiO_2_, where highly ordered layers and water contact angles exceeding 110° have frequently been reported [17,18,19,20]. However, corresponding studies on conductive delafossite oxides remain scarce. Consequently, the adsorption behaviour of phosphonic acids, thiols, and small molecule inhibitors (SMIs) on PtCoO_2_ remains largely unexplored.

In this work, we address this gap by conducting a detailed investigation into the optimisation of organic adlayer deposition on PtCoO_2_ via immersion. Specifically, we explore multiple organic passivation agents in the form of self-assembling monolayers (SAMs) and SMIs, a range of immersion times, and various surface pre-cleaning protocols, resulting in a broad experimental parameter space. By systematically evaluating these variables, we identify key factors governing the formation and uniformity of the deposited layers. It is important to note that the scope of this work is focused on understanding the formation of organic passivation layers on PtCoO_2_ and the factors governing their adsorption behaviour. By combining wetting, chemical, and morphological characterisation, this study establishes the relationship between surface termination, molecular structure, and organic layer formation on PtCoO_2_. These insights provide a foundation for the rational design of temporary passivation strategies for PtCoO_2_ processing. The stability of the deposited layers and their effectiveness in mitigating degradation under reducing environments will be the focus of future work.

## 2. Materials and Methods

### 2.1. Sample Deposition

PtCoO_2_ was grown on an in-plane c-axis-oriented (0001) Al_2_O_3_ substrate (c-sapphire). A nominal thickness of 5 nm PtCoO_2_ thin film was grown via physical vapour deposition through reactive co-sputtering from Pt and CoO targets under a flow of Ar and O_2_. During the deposition, the c-sapphire substrate was held at a temperature of 420 °C. After deposition, the samples were transferred to an Annealsys AS-One furnace for an ex situ post-deposition anneal (PDA) at 800 °C under 1 bar O_2_ for 60 min. This process was then repeated, where another 15 nm (nominal thickness) PtCoO_2_ thin film was deposited and annealed, for a total measured sample thickness of 20.0 nm.

### 2.2. Organic Layer Deposition

Four different SAMs/SMIs were compared in this study: octadecylphosphonic acid (ODPA, Sigma-Aldrich (Hoeilaart, Belgium), 97% purity), octylphosphonic acid (OPA, Sigma-Aldrich, 97% purity), 1-octadecanethiol (ODT, Sigma-Aldrich, 98% purity), and trifluoroacetic acid (TFA, Sigma-Aldrich, 99%). For the deposition of monolayer films, the immersion method was used, in which ethanol (Sigma-Aldrich, ≥99.5% purity) was used as the solvent. 1 mM solutions of ODPA, OPA, and TFA were created, whilst a concentration of 10 mM was used for the ODT, with all immersions conducted at room temperature. After immersion, all samples were rinsed in ethanol (Sigma-Aldrich, ≥99.5% purity) for a minimum of 30 s.

**Figure 1:**
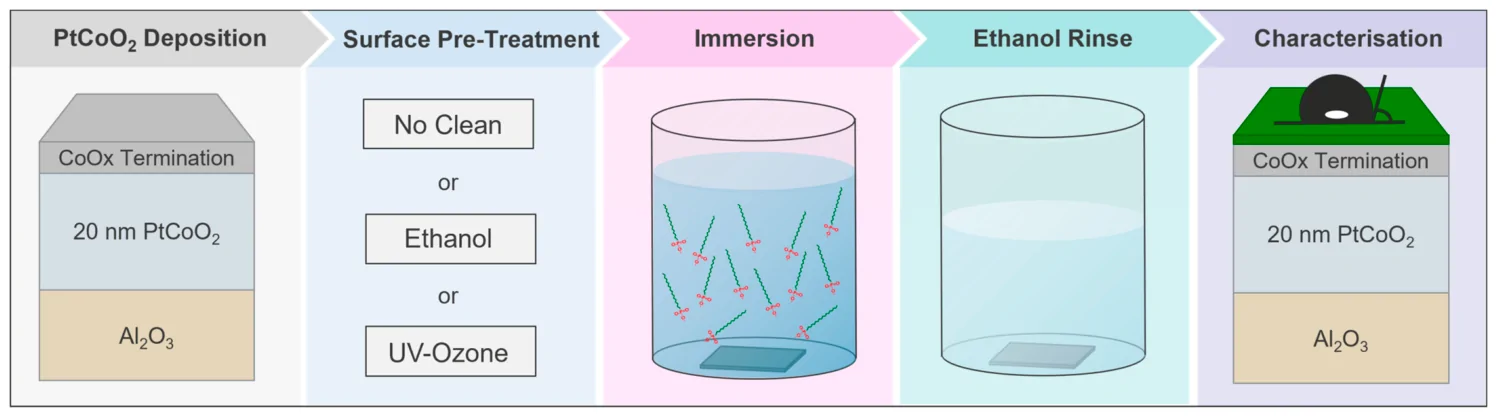
Schematic overview of the experimental workflow used to investigate adlayer formation on PtCoO_2_.

Two parameters were varied in order to optimise the deposition of the SAMs/SMIs; the first was the immersion time, where 0.5, 2, 6, 12, 24, 36, and 48 h were tested; and the second was PtCoO_2_ surface pre-cleaning prior to immersion, where no pre-clean, an ethanol rinse (Sigma-Aldrich, ≥99.5% purity), and UV-ozone clean were compared. The selected immersion times were chosen to capture both the initial adsorption regime (≤6 h), the expected monolayer growth regime (12–24 h), and extended immersion conditions (36–48 h) to evaluate whether adsorption saturation or additional surface restructuring occurred. For the ethanol rinses, the samples were submerged in room-temperature ethanol for 60 s and were dried under an N_2_ flow. For UV-ozone cleaning, a UVO-Cleaner Model 42 (Jelight Company (Irvine, CA, USA)) was used with an oxygen flow of 30 L/min and a cleaning time of 240 s. A schematic of the experimental workflow is shown in Figure 1.

### 2.3. Characterisation

Low-energy ion scattering (LEIS) measurements were conducted using an IONTOF Qtac instrument. It is equipped with a double toroidal analyser with a large solid angle of acceptance (360° azimuth) and a fixed acceptance angle (145°) of scattered ions. Two different probe ions were used: ^4^He^+^ at 3 keV were used with a dose of 1 × 10^14^ ions/cm^2^ and Ne 5 keV ions with a dose of 5 × 10^12^ ions/cm^2,^ with time-of-flight (TOF) filtering. Prior to the measurements, the surface was cleaned for 10 min using atomic oxygen generated via remote plasma.

Static water contact angle (WCA) measurements were conducted using a DataPhysics Contact Angle System (OCA) equipped with a CCD camera from Teli and a Hamilton 500 μL syringe. The drop volume was 1 μL with a dispense rate of 0.5 μL/s. The image fitting was carried out using SCA20 software with the sessile drop method and the ellipse fitting model. The WCAs represent the average of three measurements acquired at different positions on each sample, with the corresponding standard deviations shown by error bars in Figure 3. Contact angle hysteresis measurements were not performed in the present study.

X-ray photoelectron spectroscopy (XPS) was performed using a SPECS EnviroMETROS FAB system with a monochromatic Al Kα photon source. The system is equipped with an AEOLOS 150 hemispherical electron analyser and an AD-CMOS detector. Survey scans were conducted at a pass energy of 50 eV whilst high-resolution spectra were acquired at a pass energy of 10 eV. XPS fitting was conducted in CasaXPS v2.3.26.1, where the binding energy was calibrated to the bulk lattice oxide peak of the PtCoO_2_ in the O 1s at 530.7 eV. A Shirley background type was used for the C 1s and O 1s, except when there was an increasing or flat background; in this case, a linear background type was used. For the P 2p, S 2p, and F 1s, a linear background was used. For the P 2p and S 2p, the components were restrained using 0.84 eV and 1.18 eV spin–orbit splitting, respectively, and the FWHM of the doublet components were constrained to be equal where multiple peaks were constrained to have the same FWHM. A pseudo-Voigt lineshape with a Gaussian-Lorentzian ratio (denoted as GL(X) in CasaXPS) of 70:30 was used for symmetric peaks. For atomic percentage quantification, the built-in database of Scofield’s relative sensitivity factors with angle distribution correction in CasaXPS was used. Prior to analysis, a transmission function correction was applied.

Atomic force microscopy (AFM) was conducted on a Bruker Dimension Edge scanning probe microscope in tapping mode. A tetrahedral silicon Olympus OMCL-AC160TS-R3 tip was used to acquire the images, where the surface morphology was evaluated over a 2 × 2 μm^2^ scan area. Optical microscopy was performed on the same instrument as AFM using an 8× objective.

## 3. Results and Discussion

### 3.1. Surface Termination and Selection of Organic Adsorbates

**Figure 2:**
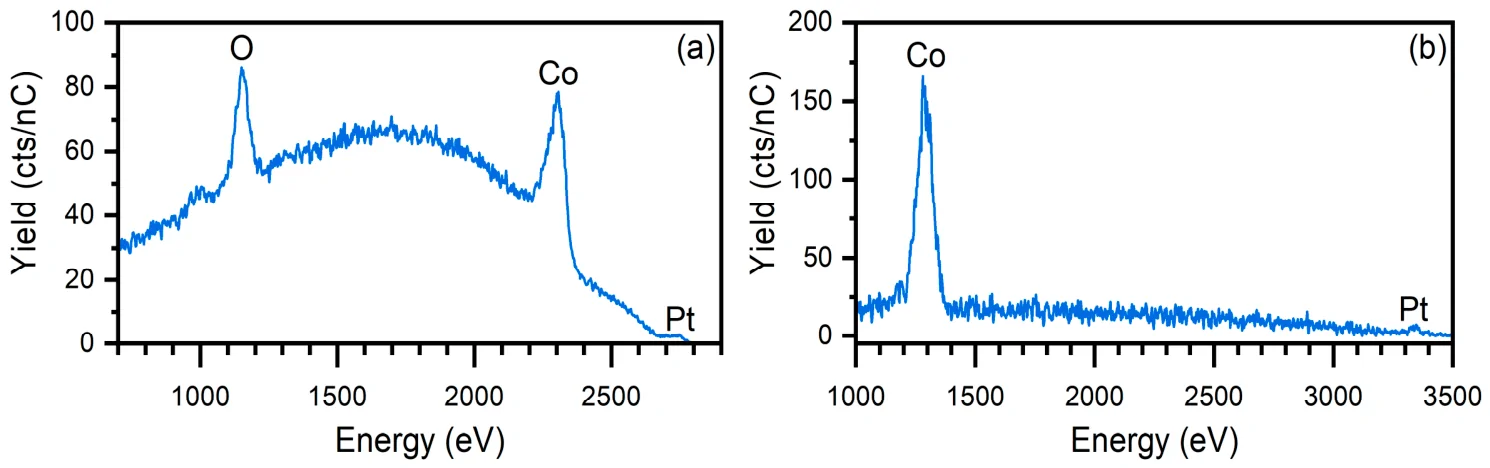
LEIS spectrum (**a**) probed using ^4^He^+^ at 3 keV ions and (**b**) probed using Ne 5 keV ions; showing dominant cobalt and oxygen signals associated with a CoOx-terminated PtCoO_2_ surface.

LEIS measurements were first conducted to establish the chemical nature of the PtCoO_2_ surface prior to organic deposition. As shown in Figure 2, the outermost atomic layer is dominated by cobalt and oxygen signals, indicating preferential segregation of a CoOx surface termination.

The composition of the terminating surface is expected to strongly influence the adsorption behaviour of organic molecules both through the availability of adsorption sites and the nature of the surface bonding interactions [21,22,23,24,25]. The identification of a CoOx-rich surface provides a framework for understanding the behaviour of the organic species investigated in this study. Phosphonic acids were selected because they are known to form stable metal-phosphonate linkages on hydroxylated metal oxide surfaces, often resulting in high surface coverage and robust attachment [20,26,27]. OPA and ODPA were chosen to isolate the effect of alkyl chain length while maintaining the same anchoring chemistry, as ODPA contains an eighteen-carbon alkyl chain, whereas OPA contains an eight-carbon chain. The longer ODPA chain is expected to promote stronger van der Waals interactions between neighbouring adsorbates, facilitating molecular ordering and reducing the formation of packing defects [28,29].

ODT was included as a contrasting adsorbate. Thiols are widely known to form highly ordered self-assembling monolayers on noble metal surfaces through metal-thiolate bond formation [30,31,32]; however, their interaction with oxide surfaces is generally weaker and often results in lower degrees of ordering [33,34]. Finally, TFA was selected as a representative small-molecule inhibitor. Unlike the long-chain SAMs, TFA lacks the extended hydrocarbon backbone required to support strong intermolecular interactions and ordered packing. Collectively, this set of molecules enables the influence of anchoring chemistry, chain length, and intermolecular interactions on adsorbate behaviour at the CoOx-terminated PtCoO_2_ surface to be systematically explored.

### 3.2. Influence of Molecular Structure and Surface Preparation on Organic Layer Formation

Water contact angle measurements were used as a rapid and sensitive probe of changes in surface wettability following organic deposition. For alkyl-terminated monolayers, increasing water contact angle is generally associated with increased hydrocarbon coverage and improved molecular organisation at the surface [35,36]. However, it should be noted that water contact angle alone cannot distinguish between complete monolayers, disordered monolayers, and multilayer structures. Consequently, the contact angle measurements are interpreted in conjunction with the XPS analysis presented later.

**Figure 3:**
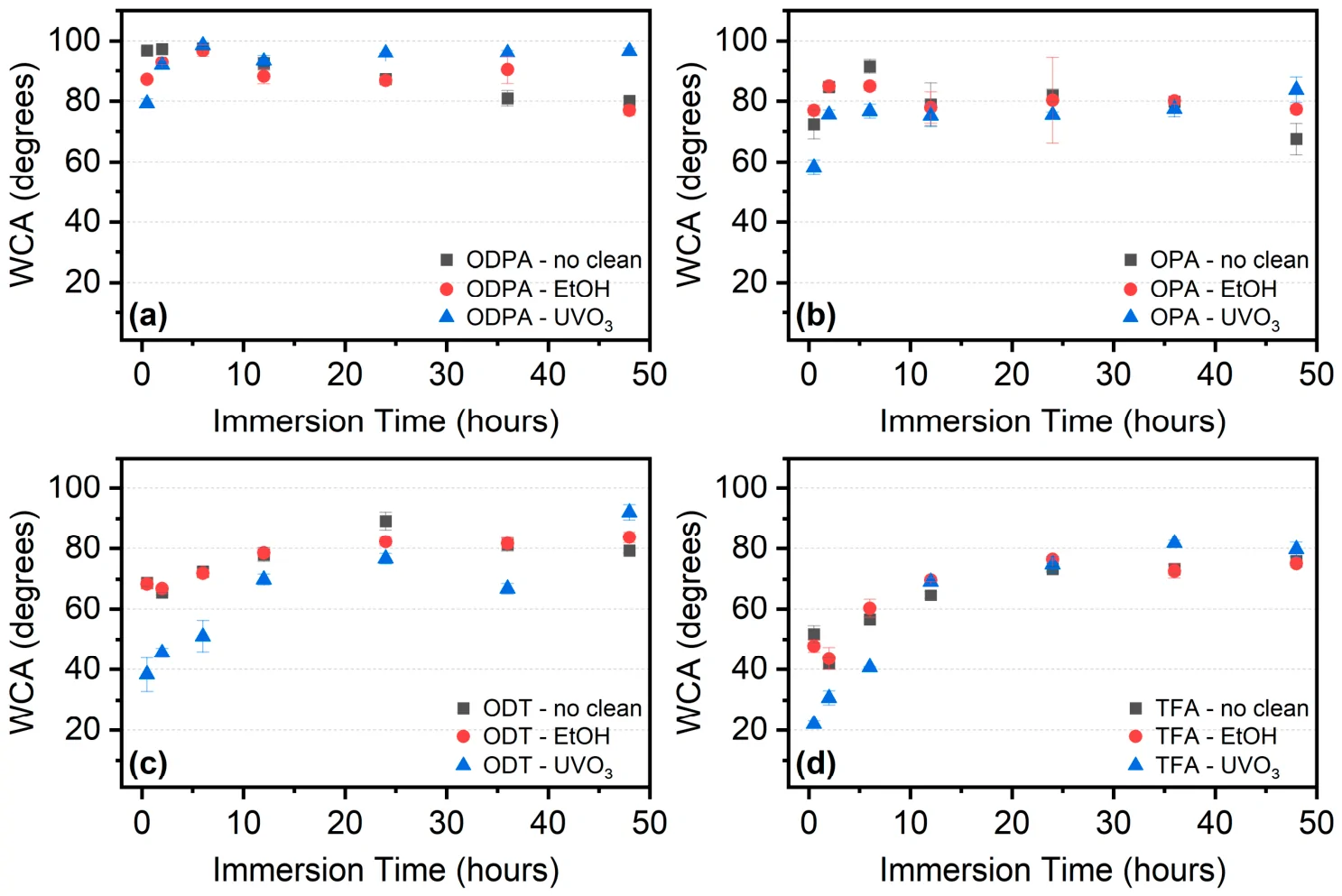
Evolution of WCA as a function of immersion time and pre-cleaning method for the deposition of (**a**) ODPA, (**b**) OPA, (**c**) ODT, and (**d**) TFA. Representative images of the WCA drop for 6 h immersion samples can be found in Figure S1.

Figure 3 summarises the evolution of contact angle with immersion time for all molecules and surface preparation conditions examined. Across all conditions, ODPA consistently produced the highest contact angles, followed by OPA and ODT, while TFA resulted in lower values. For both the phosphonic acids, contact angles approached their maximum within 2 to 6 h immersion, whereas for ODT and TFA, in most cases the maximum was reached after 24 to 36 h. The comparatively fast saturation observed for ODPA and OPA is consistent with the strong affinity of phosphonic acid towards metal oxide surfaces, where adsorption can proceed through the formation of robust phosphonate-surface bonds [17,19,37]. In contrast, the slower evolution observed for ODT and TFA suggests less favourable molecule-surface interactions, potentially resulting in slower accumulation of adsorbates at the surface before equilibrium coverage is reached [18,33,34].

The superior performance of ODPA is consistent with the combination of favourable phosphonic acid adsorption chemistry on oxide surfaces and the enhanced intermolecular interactions provided by the C18 alkyl chain. For all pre-treatment conditions, the water contact angle increased rapidly during the initial stages of immersion before approaching a plateau. Maximum contact angles of approximately 97–99° were obtained, indicating the formation of a hydrophobic organic layer. Although these values demonstrate significant modification of the PtCoO_2_ surface chemistry, they remain below the ≈105–115° values typically reported for highly ordered alkyl phosphonate monolayers on conventional oxide substrates [38,39]. This suggests that while ODPA forms the most developed organic layer among the molecules investigated, the resulting film likely contains a degree of structural disorder, incomplete coverage, or packing defects [39]. When compared to extensively studied oxide surfaces such as TiO_2_ and ZnO [17,18,19], the formation of highly ordered phosphonic acid monolayers may be intrinsically more challenging on CoOx-terminated PtCoO_2_. The complex chemistry and possible heterogeneity of the surface termination may introduce a broader distribution of adsorption environments, thereby limiting molecular ordering.

OPA exhibits broadly similar behaviour to ODPA but consistently reaches lower maximum contact angles, typically between 83 and 92°. Since OPA and ODPA share the same phosphonic acid anchoring group, the primary difference between the two molecules is alkyl chain length. The lower water contact angles obtained for OPA therefore suggest that the shorter carbon chain provides weaker lateral interactions between neighbouring adsorbates, reducing the degree of molecular organisation within the layer. This interpretation is consistent with previous studies demonstrating that increasing alkyl chain length generally promotes improved molecular ordering and lower concentrations of gauche defects in phosphonic acid monolayers [40,41].

Interestingly, the difference between ODPA and OPA is more pronounced than would be expected from differences in adsorption chemistry alone, highlighting the importance of intermolecular interactions in determining the final structure of the adsorbed layer. The results therefore suggest that both molecule-surface interactions and molecule-molecule interactions contribute significantly to organic layer formation on PtCoO_2_.

### 3.3. XPS Analysis of Phosphonic Acid Adsorption

**Figure 4:**
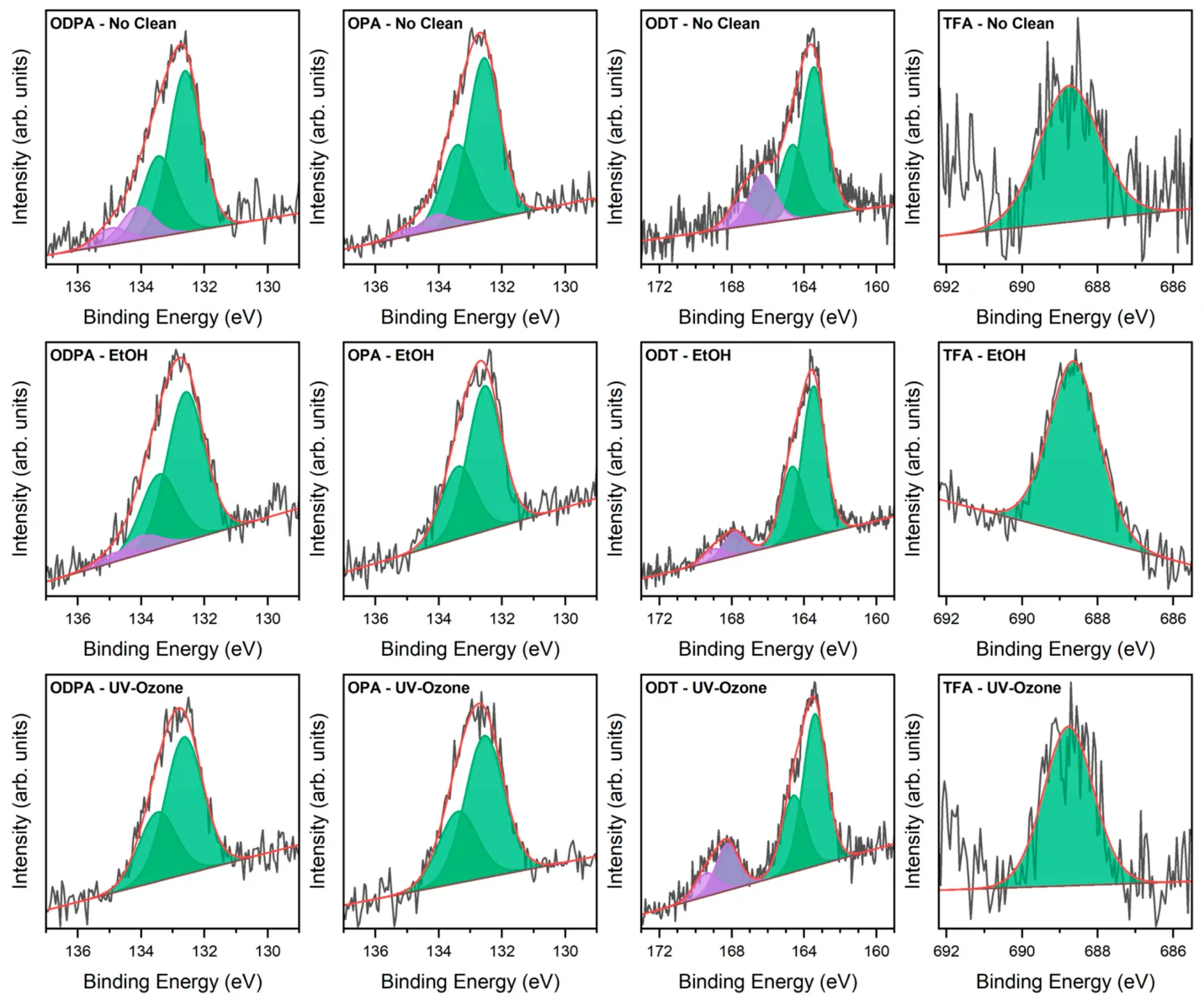
High-resolution XPS spectra of the primary elements in the functional group of interest for the SAMs/SMI (P 2p for ODPA and OPA, S 2p for ODT, and F 1s for TFA) for no clean, EtOH, and UV-ozone pre-treated PtCoO_2_.

The chemistry of the adsorbed layers was further investigated by XPS. Figure 4 compares the high-resolution spectra acquired following six hours of immersion for no clean, EtOH, and UV-ozone pre-treated PtCoO_2_.

For all three pre-treatments and for both phosphonic acids, the P 2p spectra contain a primary feature centred at 132.6 eV (green peaks). This component is attributed to chemisorbed phosphonate species, indicating that adsorption predominantly occurs through formation of phosphate-related bonding interactions with the oxide surface [42,43,44,45]. For both phosphonic acids, for the non-clean pre-treated sample and the ODPA EtOH pre-treated sample, a smaller peak at 134.0–134.1 eV (purple peaks) associated with non-deprotonated phosphonic acid species is also observed but is notably absent for UV-ozone-treated samples.

In the O 1s spectra (Figure S2), as well as the peaks associated with the bulk oxide (green peak) and surface oxide (purple peak), three additional components can be observed on the higher binding energy side (blue peaks).

For pristine PtCoO_2_, these components, located at >531.5 eV, are associated with carbon-oxygen species due to the presence of adventitious carbon (AdC) [46].

For ODPA and OPA, these peaks are associated with carbon-oxygen species from AdC, but additionally phosphorus species from the anchor group, notably the P=O at ~ 531.5 eV and P-O at ~ 532.9 eV [47,48]. Although there is not sufficient energy resolution to deconvolute these species from AdC, there is a notable downshift in the binding energy position of the primary blue peak for the phosphonic acid-based SAMs compared to TFA and ODT (Figure 4) for the samples that underwent no cleaning and EtOH pre-treatments. As TFA and ODT do not contain phosphorus species, this downshift has therefore been attributed to the presence of phosphorus-containing species. Interestingly, a lack of downshift is observed for the sample pre-treated with UV-ozone. This, paired with the fact that the higher binding energy P 2p component is not observed for samples pre-treated with UV-ozone, would suggest that the UV-ozone treatment influences the interfacial bonding environment of the phosphonic acids, likely associated with increased surface hydroxylation and/or modification of the CoOx surface chemistry. However, due to significant overlap between phosphorus-oxygen species, AdC contributions, and surface oxide species in the O 1s region, the precise origin of this behaviour cannot be established unambiguously from the data.

The influence of alkyl chain length is also reflected in the surface composition. Both ODPA and OPA exhibit comparable phosphorus concentrations for all three pre-treatments, indicating similar levels of phosphonic acid incorporation at the surface (Table 1 and Tables S1 and S2).

However, the ODPA-treated sample exhibits a substantially larger carbon contribution due to the longer hydrocarbon chain associated with each adsorbed molecule. Rather than implying a larger number of adsorbed molecules, this observation simply reflects the greater hydrocarbon content present within the ODPA layer and is consistent with the higher contact angles measured for this material. The observation of similar phosphorus concentrations despite notably different water contact angles suggests that the extent of adsorption alone cannot explain the observed wettability trends. Instead, differences in molecule packing, ordering, and the presentation of the terminal alkyl groups at the interface are likely important contributors to the final surface properties. This indicates that achieving a high adsorption density is not sufficient by itself; the organisation of the adsorbed layer must also be considered when evaluating the effectiveness of organic passivation strategies.

Taken together, the WCA and XPS results indicate that phosphonic acids are the most effective anchoring chemistry investigated for the CoOx-terminated PtCoO_2_ surface, with ODPA producing the most hydrophobic and chemically well-defined organic layer.

### 3.4. Surface Morphology of Deposited Organic Layers

**Figure 5:**
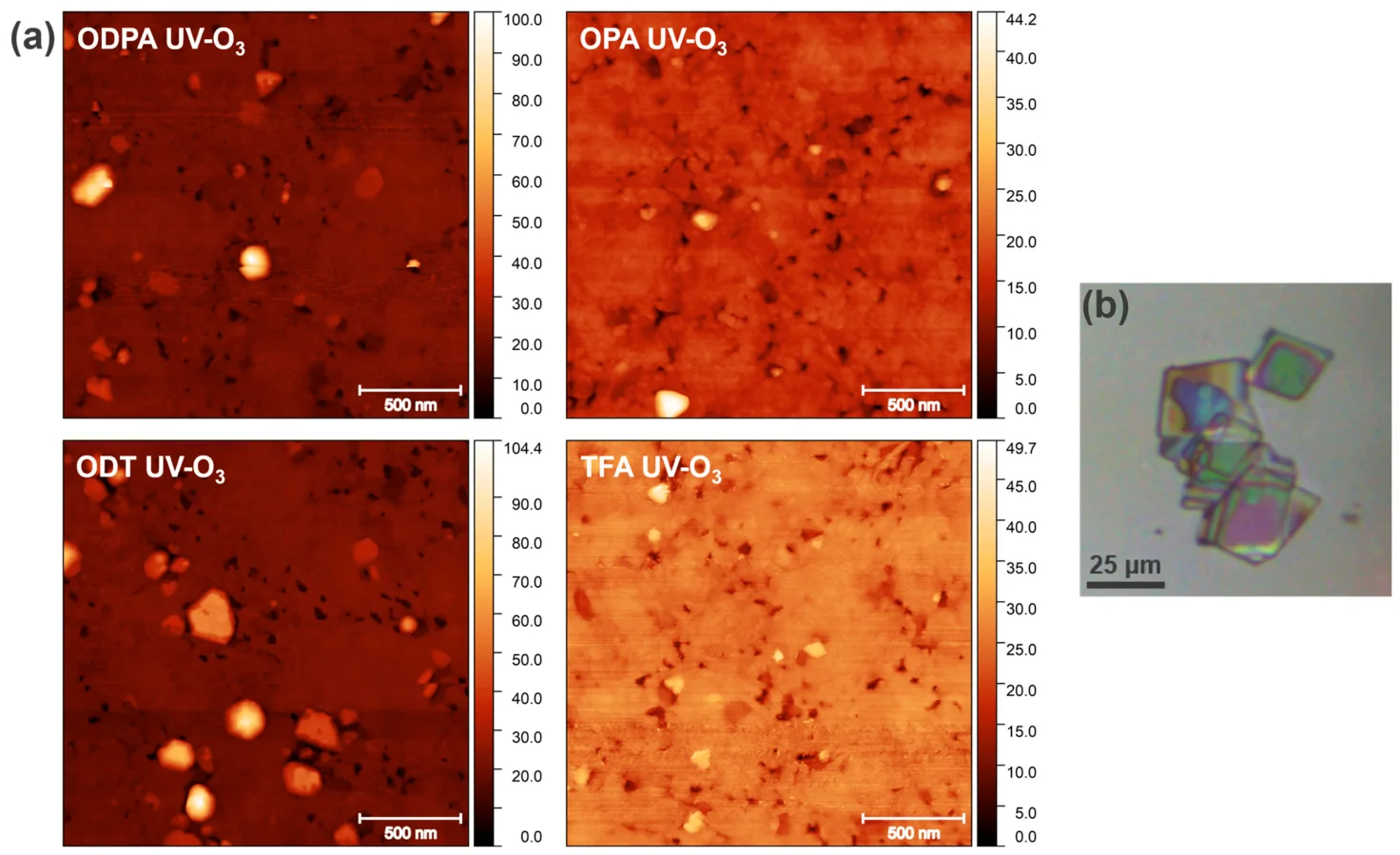
(**a**) 2 × 2 μm AFM images of the PtCoO_2_ surface which underwent UV-ozone pre-cleaning, after the deposition of SAMs and SMI via immersion for 12 h. (**b**) Optical microscopy image (8× objective) of a crystallite observed on the PtCoO2 sample that underwent UV-ozone pre-cleaning and 12 h immersion in the ODT deposition solution.

To further investigate the influence of molecular structure and surface preparation on organic layer formation, AFM measurements were conducted following 12 h immersion. Although the thickness of a self-assembled monolayer is substantially below the dimensions of the topographic features observed in AFM, the measurements provide insight into mesoscale surface morphology resulting from the deposition process. Representative AFM images are shown in Figure 5a and Figures S4 and S5, whilst the extracted roughness parameters are summarised in Table 2 and Table S3.

For the no-clean and EtOH-cleaned samples, relatively similar root-mean-square (RMS) roughness (Rq) values of approximately 3 nm were observed for all organic species. More substantial differences emerged following UV-ozone treatment, where the resulting morphology exhibited a stronger dependence on the deposited organic. In particular, ODPA and ODT exhibited significantly increased roughness, with Rq values of 5.79 and 7.67 nm, respectively, compared to 2.51 and 2.97 nm for OPA and TFA. A similar trend was observed in the peak-to-valley measurements, where ODPA and ODT displayed the largest vertical topographic variations, reaching approximately 100 and 104 nm, respectively.

The increased roughness observed for ODPA after UV-ozone treatment suggests that the modified surface promotes the formation of larger adsorbate domains or preferential accumulation of organic material at specific surface sites. This behaviour is consistent with the strong affinity of phosphonic acids for metal oxide surfaces and may reflect enhanced adsorption at hydroxylated surface species generated during UV-ozone exposure.

In addition to the increased topological variation, the ODPA on UV-ozone pre-treated PtCoO_2_ also exhibits the highest water contact angles among all the molecules investigated. Although increased roughness may contribute to the observed wettability through morphology-related effects, the data suggest that roughness alone is unlikely to fully explain the increase in WCA, and therefore changes in adsorption behaviour may also play a role. Notably, ODT exhibits an even greater surface roughness following UV-ozone treatment (Rq = 7.67 nm) yet does not achieve the correspondingly higher WCA. This observation indicates that the chemical nature and organisation of the adsorbed layer play an important role in determining the wetting behaviour. For example, differences in phosphonic acid binding configuration could influence molecular packing and orientation, with variations in the extent of mono-, bi-, or tridentate attachment potentially affecting the exposure of terminal alkyl chains at the surface and therefore the measured contact angle. Nevertheless, the high contact angle indicates that the formation of localised surface features does not prevent the development of a relatively hydrophobic organic layer.

In contrast, OPA exhibited substantially lower roughness and peak-to-valley values under all surface preparation conditions. Since OPA and ODPA possess the same phosphonic acid anchor group, the primary distinction between these molecules is alkyl chain length. The reduced topographic variation observed for OPA is therefore consistent with the weaker intermolecular interactions expected for the shorter alkyl chain, which may limit the formation of larger organised domains on the surface. This observation aligns with the lower water contact angles obtained for OPA relative to ODPA and further supports the importance of chain length in determining the final structure of the adsorbed layer.

The most pronounced morphological differences were observed for ODT. Following UV-ozone treatment, ODT exhibited both the highest roughness and the largest peak-to-valley values measured in this study. AFM images additionally revealed the presence of large protruding surface features not observed to the same extent for the phosphonic acid systems. Complementary optical microscopy (Figure 5b) performed after immersion revealed the presence of micron-scale faceted crystallites on ODT-treated samples. The appearance of these crystalline features, combined with the elevated carbon and sulphur concentrations observed by XPS, suggests that a fraction of the deposited ODT exists as aggregated or crystallised adsorbate domains rather than exclusively as a uniform self-assembled monolayer. This interpretation provides a possible explanation for the apparent discrepancy between the relatively high carbon and sulphur concentrations detected by XPS and the lower-than-expected water contact angles obtained for ODT. While substantial amounts of ODT-derived material are present at the surface, a portion of this material appears to be associated with heterogeneous surface structures that do not contribute effectively to the formation of a densely packed and highly ordered hydrophobic interface.

TFA exhibited the smallest overall changes in surface morphology. Both the roughness and peak-to-valley values remained relatively low across all pre-treatment conditions, and no evidence for the formation of large surface aggregates was observed. These observations are consistent with the XPS measurements, which indicated only limited retention of fluorinated species at the surface, and with the comparatively low water contact angles measured for TFA. Together, these results suggest that TFA does not produce a substantial or highly organised surface layer on PtCoO_2_ under the deposition conditions investigated.

Overall, the AFM results demonstrate that the choice of both anchoring chemistry and surface preparation strongly influences the resulting surface morphology. The phosphonic acids generally produce relatively uniform surfaces, with ODPA exhibiting the greatest degree of hydrophobic surface modification. In contrast, ODT displays evidence of aggregate and crystallite formation, highlighting that a high surface concentration of adsorbed material does not necessarily correspond to the formation of a homogeneous and well-ordered monolayer. These findings complement the WCA and XPS measurements and further illustrate the critical role of molecule-surface interactions in governing organic layer formation on CoOx-terminated PtCoO_2_.

### 3.5. Adsorption of Octadecanethiol on PtCoO_2_

The behaviour of ODT differs notably from that of the phosphonic acids. Despite producing maximum water contact angles comparable to those obtained with OPA under some conditions, the XPS data suggest a fundamentally different adsorption mechanism.

For all pre-treatment methods, two sulphur-containing species are observed in the S 2p spectrum (Figure 4), where the dominant component at 163.5 eV (green peaks) is characteristic of molecular thiol species [49,50]. The dominance of this unbound thiol component suggests that a significant fraction of ODT does not form conventional thiolate bonding at the PtCoO_2_ surface. The secondary species (purple peaks) are associated with oxidised sulphur for all three pre-treatments; however, the differing binding-energy positions are suggestive of oxidised sulphur in different oxidation states. For the no-clean sample, the secondary peak is observed at 166.4 eV; for EtOH, it is observed at 167.8 eV; and for UV-ozone, it is observed at 168.2 eV. This progressive shift towards higher binding energies suggests the formation of increasingly oxidised sulphur species with increased surface oxidation [51,52,53,54,55,56]. A plausible explanation is that the UV-ozone treatment generates a more oxygen-rich and hydroxylated CoOx surface, promoting partial oxidation of adsorbed ODT molecules. The oxidation of the anchor group may be beneficial and promote surface adsorption of ODT by providing a bonding mechanism that more closely resembles that of phosphonic acids. The observation that the dominant thiol component remains largely unchanged indicates that only a fraction of the deposited ODT undergoes a chemical transformation. Together with AFM and optical microscopy, which indicate the presence of heterogeneous surface aggregates, these results suggest that ODT adsorption on PtCoO_2_ proceeds through a complex combination of molecular adsorption and oxidative surface reactions rather than the formation of a conventional thiolate self-assembled monolayer.

This behaviour is consistent with expectations based on the CoOx-rich surface termination identified by LEIS. In contrast to Au and other metallic surfaces, where alkanethiols typically form densely packed thiolate self-assembled monolayers [32,34,50], the PtCoO_2_ surface exhibits a predominance of molecular thiol species together with oxidised sulphur species, indicating fundamentally different adsorption behaviour. The coexistence of these species suggests a heterogeneous interfacial structure involving both weakly bound and chemically transformed sulphur-containing adsorbates, highlighting the strong influence of the CoOx-rich oxide termination on the adsorption chemistry.

The effect of surface preparation is particularly pronounced for ODT. Whereas neither clean nor ethanol-cleaned samples exhibit relatively similar behaviour, UV-ozone treatment initially suppresses the increase in contact angle before ultimately producing the highest contact angle observed for ODT after prolonged immersion. This behaviour contrasts with reports for metallic copper surfaces [16] and may reflect differences in the chemistry and stability of the oxide layers generated during UV-ozone treatment.

### 3.6. Adsorption of Trifluoroacetic Acid

TFA exhibits the weakest overall surface modification among the adsorbates investigated. Consistently lower contact angles were measured across all processing conditions, indicating limited formation of a hydrophobic surface layer.

This observation is supported by XPS, which shows only a low fluorine concentration (Table 1). Furthermore, no significant CF_2_ or CF_3_ contributions are observed in the C 1s region (Figure S3) except for the EtOH pre-treated sample, where only a minor contribution is detected (~1%) [57,58,59]. The XPS limit of detection (LOD) for fluorine was estimated following the methodology of Shard [60], where an approximate value of 0.6 at.% was obtained for a Pt-containing matrix under the acquisition conditions employed here. While this estimate is based on a homogeneous bulk distribution of fluorine and therefore should be regarded as approximate for a surface-adsorbed species such as TFA, it nevertheless provides a useful upper-bound assessment of detectability. The measured fluorine concentration (~0.3 at.%) together with the absence of discernible CF_2_/CF_3_ contributions in the C 1s spectrum indicates that only trace quantities of TFA remain on the surface and does not support the formation of a dense fluorinated adlayer.

The small F 1s peak which is observed is at a position of 688.6–688.8 eV, which is associated with organic fluorine rather than metal fluoride, which is observed at a significantly lower binding energy of ~685 eV [61,62,63]. The absence of metal fluoride suggests that TFA does not strongly fluorinate or substantially etch the CoOx-terminated PtCoO_2_ surface under the conditions investigated.

Instead, interaction of TFA with the CoOx-terminated surface is expected to occur primarily through acid-base reactions and ligand exchange at surface cobalt oxide sites, yielding adsorbed cobalt trifluoroacetate species [64,65]. Such surface complexation is consistent with the presence of a weak F 1s signal observed. Although a distinct CF_3_ contribution is largely absent from the C 1s spectra, this does not necessarily rule out the presence of a surface trifluoroacetate species, since at very low coverages the corresponding carbon signal may fall below the detection limit or be masked by the dominant AdC contribution, while fluorine remains detectable in the F 1s region due to its comparatively low background signal. The low fluorine coverage may therefore arise from incomplete adsorption, desorption during subsequent handling, or formation of only a sparsely populated surface trifluoroacetate layer.

Overall, the combination of the low fluorine concentration, the absence of significant CF_2_/CF_3_ spectral features, and the modest change in wettability indicates that TFA only weakly interacts with the CoOx-terminated PtCoO_2_ surface. Rather than forming a dense passivation organic overlayer, TFA appears to act primarily as a surface-coordinating ligand, resulting in only limited modification of the surface chemistry and wetting behaviour.

### 3.7. Implications for Organic Passivation of PtCoO_2_

The combined LEIS, WCA, and XPS results identify a clear dependence of adsorption behaviour on both anchoring chemistry and molecular structure. The CoOx-rich surface termination of PtCoO_2_ strongly favours phosphonic acid adsorption, resulting in more chemically robust and hydrophobic surface layers than those obtained using either thiol- or carboxylic-acid-based adsorbates.

However, even the best-performing system, ODPA, produced maximum contact angles below those commonly reported for highly ordered alkyl monolayers on conventional oxide surfaces. This observation suggests that achieving fully ordered, defect-free monolayers on PtCoO_2_ may be challenging under the conditions examined in this study. Possible contributing factors include the surface structure of PtCoO_2_, the chemistry of the CoOx termination layer, limitations associated with immersion deposition, and the choice of solvent and processing conditions.

Consequently, while ODPA has been identified as the most promising candidate among the molecules investigated here, further optimisation through refinement of surface preparation procedures, solvent selection, solution composition, and deposition conditions may enable additional improvements in film quality. These findings establish a foundation for future studies focused on evaluating the effectiveness of optimised organic layers as temporary passivation coatings during processing in reducing environments.

Beyond identifying ODPA as the most promising adsorbate, this work also provides new insight into how conductive delafossite oxide surfaces interact with different classes of organic molecules. While phosphonic acids, thiols, and carboxylic acids have all been widely investigated on conventional metals and oxides, their comparative adsorption behaviour on PtCoO_2_ had not previously been established. The results demonstrate that the CoOx-rich surface termination strongly favours phosphonic acid adsorption while limiting the formation of conventional thiol-based self-assembled monolayers. These findings therefore provide important design guidelines for selecting adsorption chemistries for surface modification of PtCoO_2_ and potentially other conductive oxide interconnect materials.

## 4. Conclusions

In this work, the deposition of organic adlayers on PtCoO_2_ was systematically investigated to establish the key factors governing surface functionalisation of this emerging conductive delafossite oxide. By combining LEIS, water contact angle, XPS, and AFM analysis, the influence of anchoring chemistry, molecular structure, immersion time, and surface pre-treatment on organic layer formation was evaluated.

LEIS measurements confirmed that the PtCoO_2_ surface is terminated by a CoOx-rich layer, which strongly influences adsorption behaviour. Among the molecules investigated, phosphonic acids exhibited the most favourable interactions with the oxide surface. ODPA consistently produced the highest water contact angles, reaching approximately 97–99°, and exhibited clear evidence of phosphonate bonding by XPS, indicating the formation of the most developed organic layer. In comparison, OPA produced lower WCAs of approximately 83–92°, demonstrating that alkyl chain length plays an important role in determining the final surface properties. The longer C18 chain of ODPA promotes stronger intermolecular interactions and enhanced hydrophobicity relative to the shorter C8 chain of OPA. In contrast, ODT displayed evidence of heterogeneous adsorption, oxidation of sulphur species, and aggregate formation, while TFA showed only trace fluorine concentrations (~0.3 at.%), demonstrating limited retention at the surface and subsequent minimal modification of wettability.

The results further demonstrate that surface preparation significantly affects organic layer formation. UV-ozone treatment altered the adsorption behaviour of both phosphonic acids and ODT, resulting in increased roughness for ODPA (Rq = 5.79 nm) and ODT (Rq = 7.67 nm) compared with the corresponding no-clean and ethanol-cleaned samples. However, despite these changes, ODPA remained the most effective adsorbate across all pre-treatment conditions. Immersion time studies showed that ODPA and OPA reached near-saturation values within 2–6 h, whereas ODT and TFA generally required 24–36 h to reach maximum wettability changes, indicating slower adsorption kinetics and less favourable molecule-surface interactions.

Overall, this study identifies ODPA as the most promising organic passivation candidate for PtCoO_2_ among the molecules evaluated and establishes a framework linking surface termination, molecular structure, and adsorption behaviour on delafossite oxide surfaces. These findings provide important design guidelines for the development of organic passivation strategies for PtCoO_2_ and related oxide conductors. Future work will focus on assessing the stability of the optimised organic layers and their effectiveness in mitigating PtCoO_2_ degradation during exposure to reducing process environments relevant to semiconductor device integration.

## Figures and Tables

**Table 1:** Atomic percentage (at. %) composition extracted from high-resolution XPS spectra of the C 1s, O 1s, Co 2p_3/2_, Pt 4f, and the primary element in the functional group of interest for OPDA, OPA, ODT, and TFA when PtCoO_2_ has not undergone any pre-treatment.

SAMs/SMI	C 1s (at. %)	O 1s (at. %)	Co 2p_3/2_ (at. %)	Pt 4f (at. %)	Functional Group (at. %)
ODPA	40.6	33.8	12.1	11.5	2.0
OPA	26.0	42.9	16.0	13.4	1.9
ODT	51.4	27.0	9.1	9.4	2.7
TFA	19.8	45.8	18.5	15.6	0.3

**Table 2:** RMS roughness extracted from AFM after 12 h immersion.

SAMs/SMI	No Clean Rq (nm)	EtOH Rq (nm)	UV-Ozone Rq (nm)
ODPA	3.13	3.08	5.79
OPA	3.19	2.85	2.51
ODT	3.15	3.04	7.67
TFA	2.80	3.10	2.97

## Data Availability

The data that supports these findings will be available in Zenodo at https://doi.org/10.5281/zenodo.21884677, following an embargo from the date of publication to allow for the commercialisation of research findings.

## Supplementary Materials

The following supporting information can be downloaded at https://www.mdpi.com/article/10.3390/nano16201296/s1, Figure S1: Representative WCA images of the PtCoO_2_ surface which underwent either no clean, ethanol pre-cleaning, or UV-ozone precleaning, after the deposition of ODPA, OPA, ODT, and TFA via immersion for 6 h; Figure S2: High-resolution O1 s spectra of ODPA, OPA, ODT, and TFA for no clean, EtOH, and UV-ozone pre-treated PtCoO_2_; Figure S3: High-resolution C1 s spectra of ODPA, OPA, ODT, and TFA for no clean, EtOH, and UV-ozone pre-treated PtCoO_2_; Table S1: Atomic percentage (at. %) composition extracted from high-resolution XPS spectra of the C 1s, O 1s, Co 2p_3/2_, Pt 4f, and the primary element in the functional group of interest for OPDA, OPA, ODT, and TFA, when PtCoO_2_ has been pre-treated with EtOH; Table S2: Atomic percentage (at. %) composition extracted from high-resolution XPS spectra of the C 1s, O 1s, Co 2p_3/2_, Pt 4f, and the primary element in the functional group of interest for OPDA, OPA, ODT, and TFA, when PtCoO2 has been pre-treated with UV-ozone; Figure S4: 2 × 2 μm AFM images of the PtCoO_2_ surface which had not undergone any pre-cleaning, after the deposition of self-assembling monolayers (SAMs) and small molecule inhibitor (SMI) via immersion for 12 h; Figure S5: 2 × 2 μm AFM images of the PtCoO_2_ surface which underwent an ethanol pre-cleaning, after the deposition of SAMs and SMI via immersion for 12 h; Table S3: Maximum peak-to-valley height extracted from AFM after 12 h immersion. Figure S6: Uncropped optical microscopy image (8x objective) of Figure 5b of a crystallite observed on the PtCoO_2_ sample that underwent a UV-ozone pre-clean and 12 h immersion in ODT deposition solution.

## Abbreviations

The following abbreviations are used in this manuscript:

SAM	Self-assembling monolayer
SMI	Small molecule inhibitor
WCA	Water contact angle
XPS	X-ray photoelectron spectroscopy
LEIS	Low-energy ion scattering
AFM	Atomic force microscopy
ODPA	Octadecylphosphonic acid
OPA	Octylphosphonic acid
ODT	1-octadecanethiol
TFA	Trifluoroacetic acid
EtOH	Ethanol
UV	Ultra-violet
