# Supplementary material for: Understanding Organic Adlayer Formation on CoOx-Terminated PtCoO2
Source: Nanomaterials (Basel). 2026 Oct 13;16(20):1296. doi: 10.3390/nano16201296 (PMC13708522; doi:10.3390/nano16201296)
Supplement: Supplementary file 1 [file nanomaterials-16-01296-s001.zip › nanomaterials-4526695-supplementary.pdf]

Supplementary Material

Understanding Organic Adlayer Formation on CoOx-Terminated $PtCoO_2$

Kerry Hazeldine [1,*], Jean-Philippe Soulié [1], Marko Vehkamäki [2], Mikko Ritala [2], Christoph Adelmann [1] and Silvia Armini [1]

[1] IMEC, 3001 Leuven, Belgium
[2] Department of Chemistry, University of Helsinki, 00560 Helsinki, Finland
* Correspondence: kerry.hazeldine@imec.be

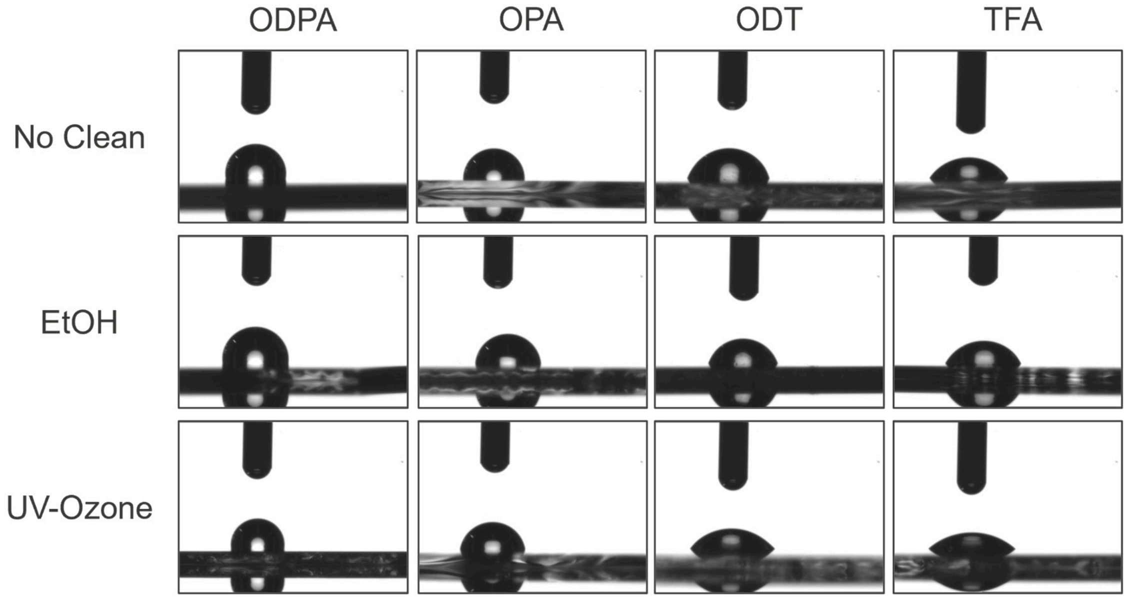

Figure S1. Representative WCA images of the $PtCoO_2$ surface which underwent either no clean, ethanol pre-cleaning, or UV-ozone precleaning, after the deposition of ODPA, OPA, ODT, and TFA via immersion for 6 hours.

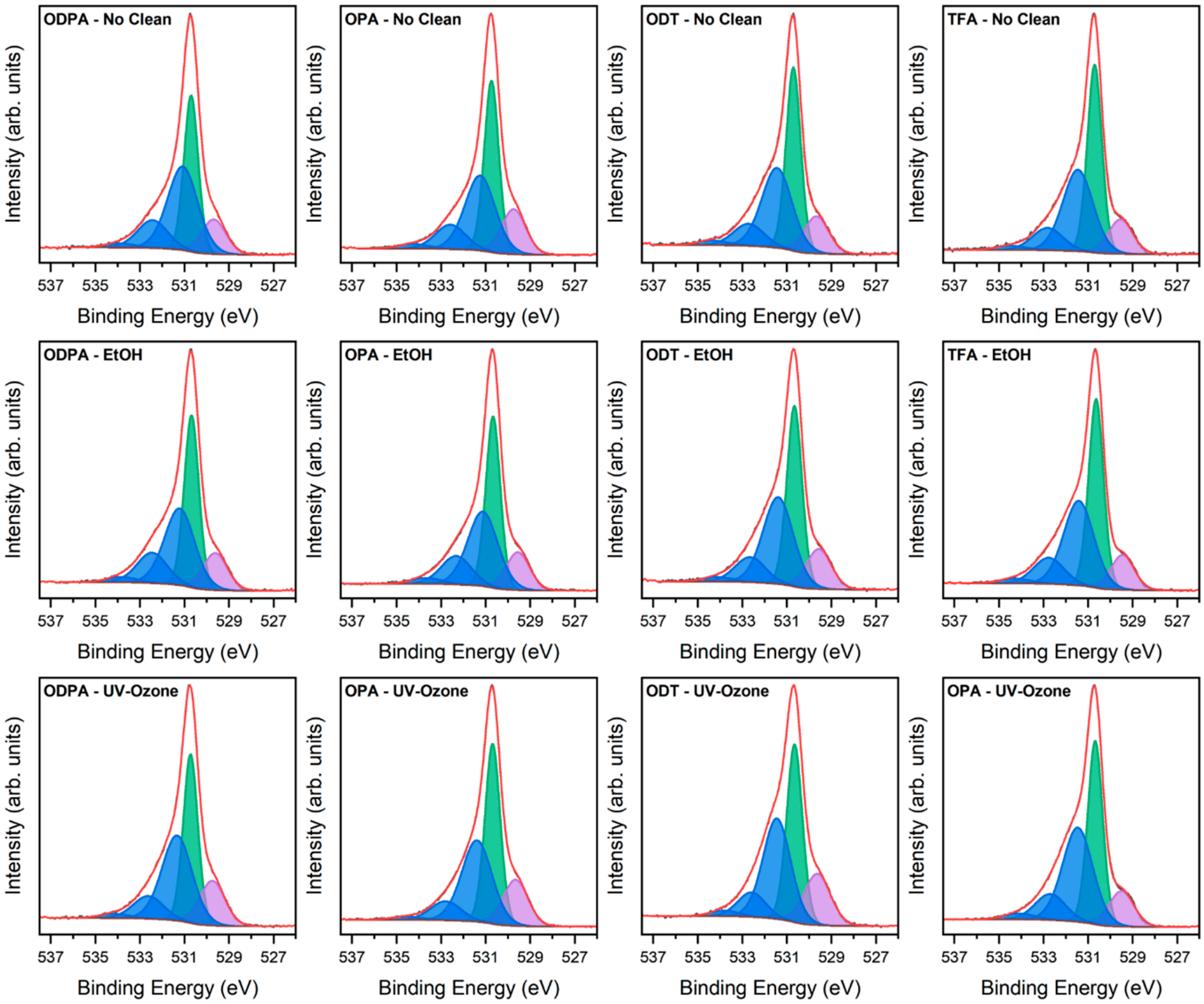

Figure S2. High-resolution O1 s spectra of ODPA, OPA, ODT, and TFA for no clean, EtOH, and UV-ozone pre-treated $PtCoO_2$.

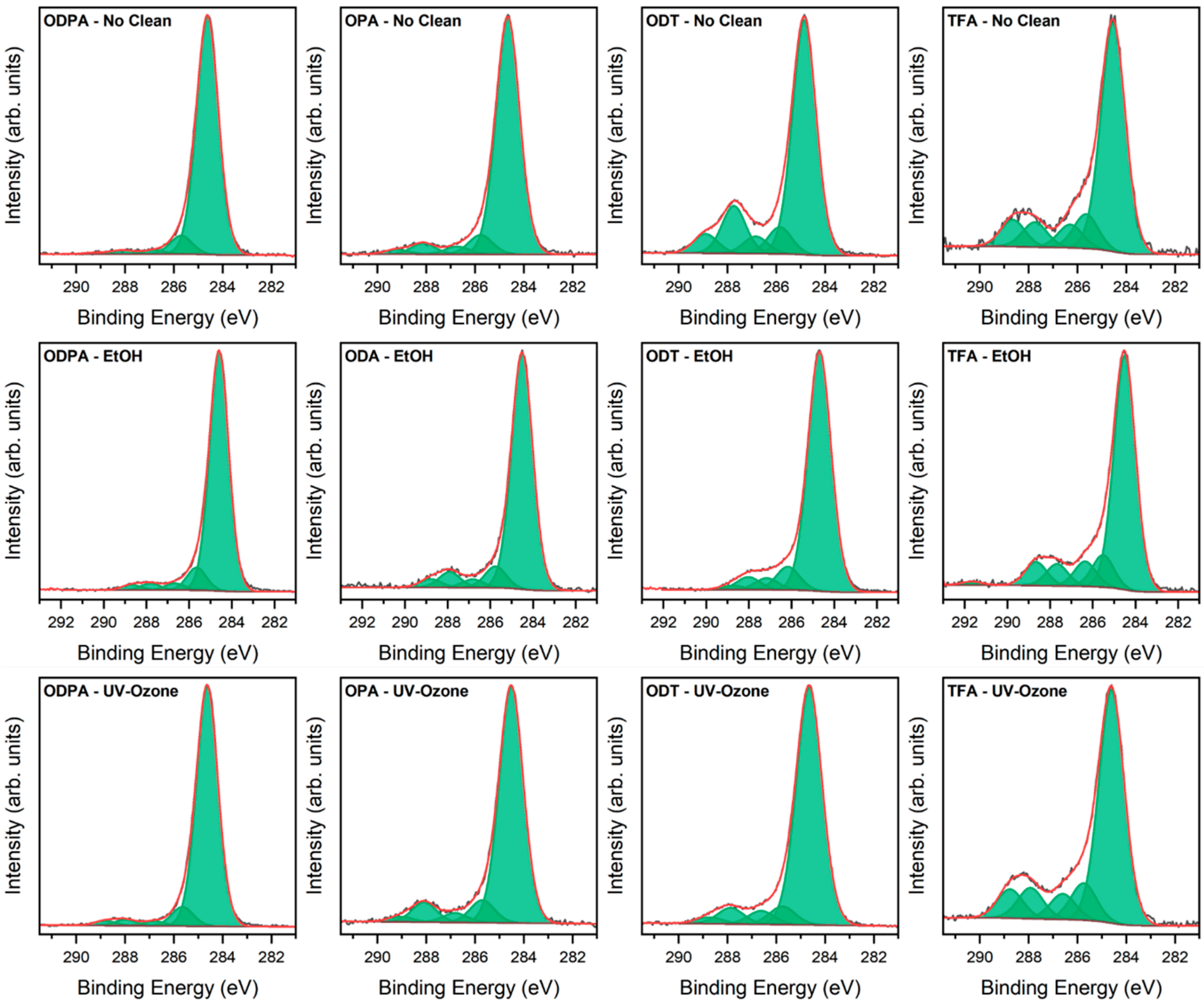

Figure S3. High-resolution C1 s spectra of ODPA, OPA, ODT, and TFA for no clean, EtOH, and UV-ozone pre-treated $PtCoO_2$.

Table S1. Atomic percentage (at. %) composition extracted from high-resolution XPS spectra of the C 1s, O 1s, Co $2p_{3/2}$, Pt 4f, and the primary element in the functional group of interest for OPDA, OPA, ODT, and TFA, when PtCoO2 has been pre-treated with EtOH.

	C 1s (at. %)	O 1s (at. %)	Co $2p_{3/2}$ (at. %)	Pt 4f (at. %)	Head Group Element (at. %)
**ODPA**	39.7	35.2	12.2	11.6	1.3
**OPA**	22.6	44.8	16.9	14.5	1.2
**ODT**	45.3	32.3	10.4	10.1	1.9
**TFA**	26.0	45.6	15.6	14.1	0.7

Table S2. Atomic percentage (at. %) composition extracted from high-resolution XPS spectra of the C 1s, O 1s, Co $2p_{3/2}$, Pt 4f, and the primary element in the functional group of interest for OPDA, OPA, ODT, and TFA, when PtCoO2 has been pre-treated with UV-ozone.

	C 1s (at. %)	O 1s (at. %)	Co $2p_{3/2}$ (at. %)	Pt 4f (at. %)	Functional Group Element (at. %)
**ODPA**	41.3	34.7	11.8	10.8	1.4
**OPA**	23.9	44.9	16.6	13.3	1.3
**ODT**	42.2	34.8	11.6	9.7	1.7
**TFA**	22.0	46.7	16.8	14.2	0.3

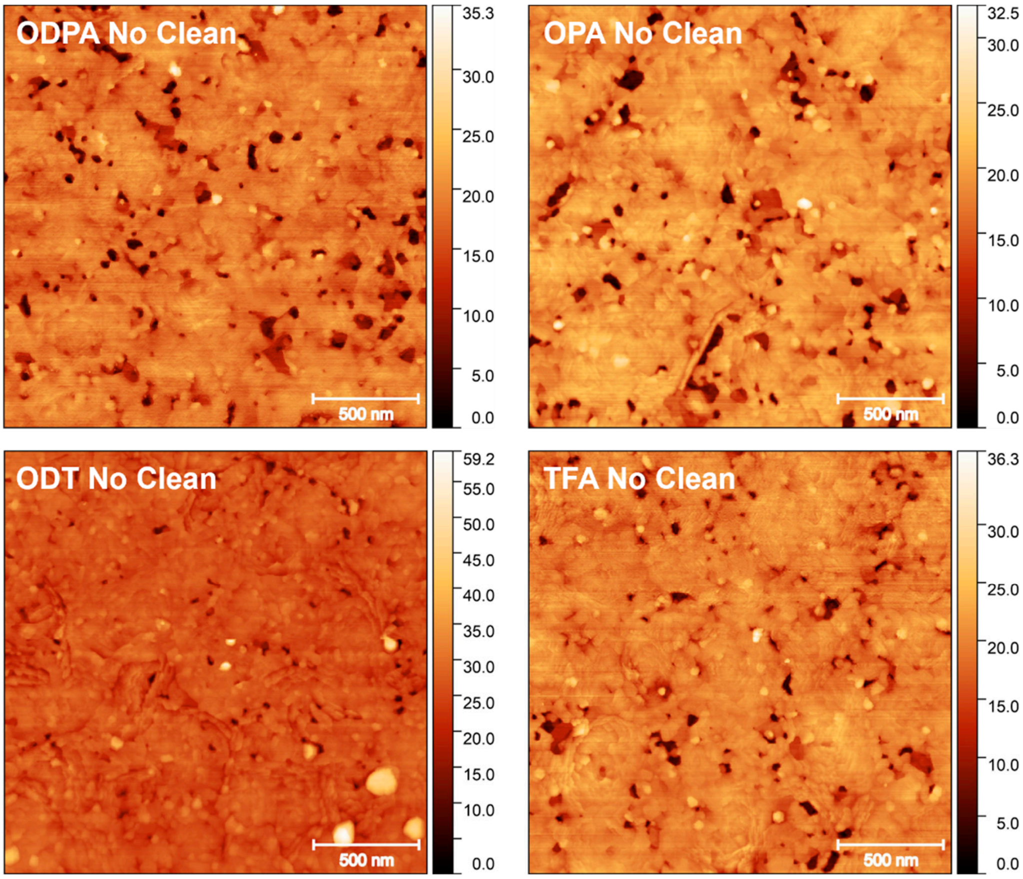

Figure S4. 2 x 2 μm AFM images of the $PtCoO_2$ surface which had not undergone any pre-cleaning, after the deposition of self-assembling monolayers (SAMs) and small molecule inhibitor (SMI) via immersion for 12 hours.

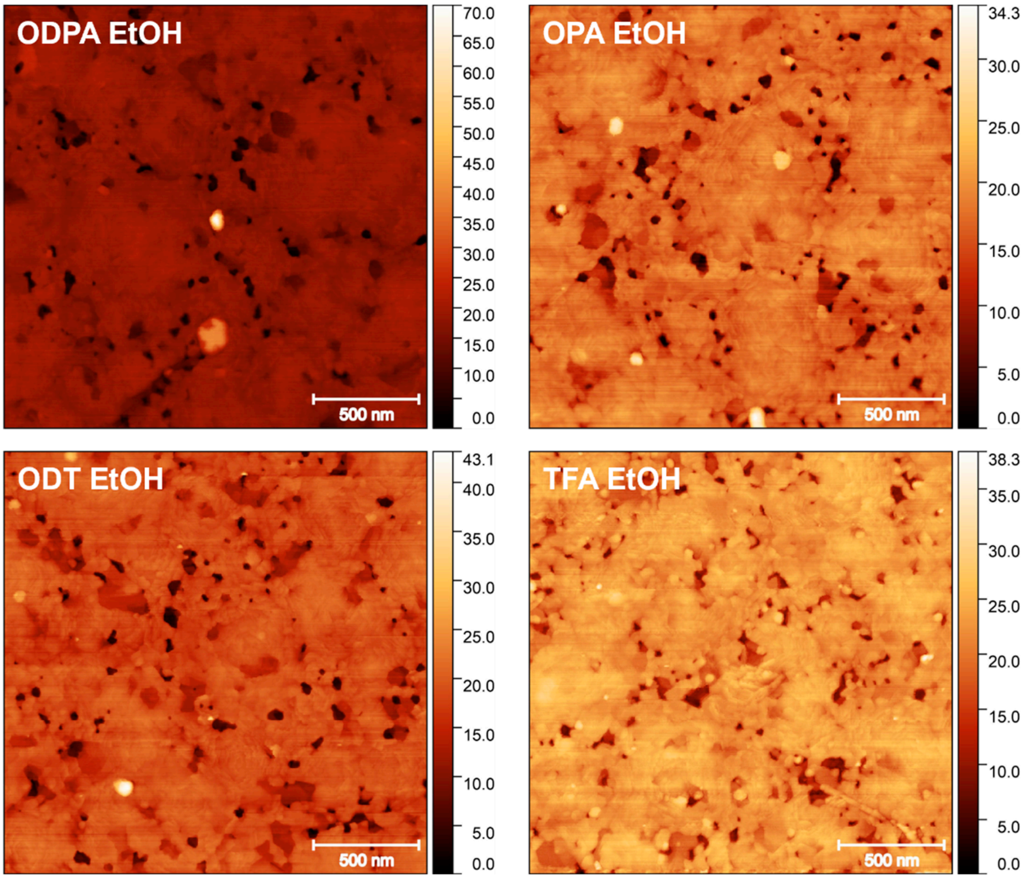

Figure S5. 2 x 2 μm AFM images of the $PtCoO_2$ surface which underwent an ethanol pre-cleaning, after the deposition of SAMs and SMI via immersion for 12 hours.

Table S3. Maximum peak-to-valley height extracted from AFM after 12 hour immersion.

	No Clean<br>Peak to Valley (nm)	EtOH<br>Peak to Valley (nm)	UV-Ozone<br>Peak to Valley (nm)
**ODPA**	35.32	69.99	99.98
**OPA**	32.46	34.34	44.23
**ODT**	59.20	43.10	104.4
**TFA**	36.26	38.34	49.68

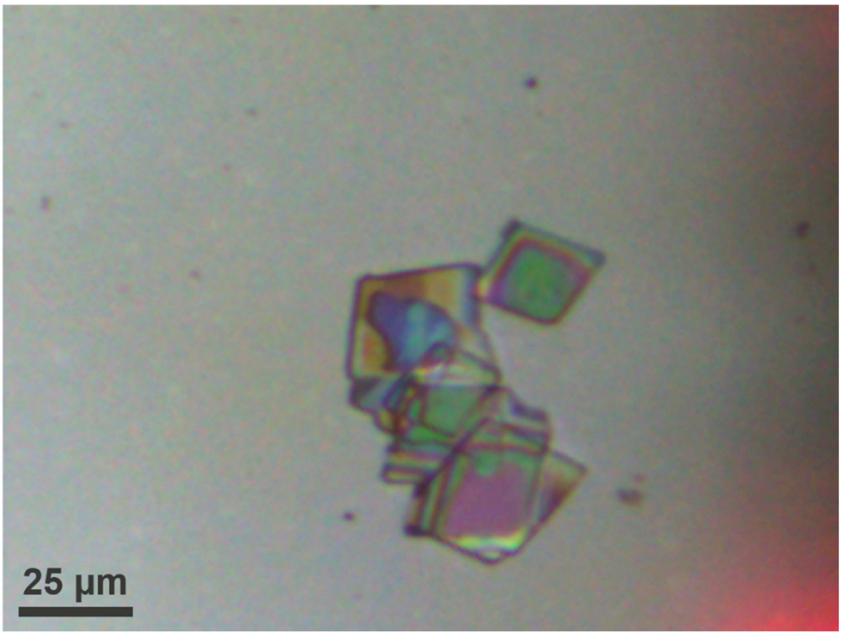

Figure S6. Uncropped Optical microscopy image (8x objective) of Figure 5b of a crystallite observed on the $PtCoO_2$ sample that underwent a UV-ozone pre-clean and 12 hours immersion in ODT deposition solution.
